# The Complex Vaginal Flora of West African Women with Bacterial Vaginosis

**DOI:** 10.1371/journal.pone.0025082

**Published:** 2011-09-20

**Authors:** Jacques Pépin, Sylvie Deslandes, Geneviève Giroux, François Sobéla, Nzambi Khonde, Soumaila Diakité, Sophie Demeule, Annie-Claude Labbé, Nathalie Carrier, Eric Frost

**Affiliations:** 1 Department of Microbiology and Infectious Diseases, Université de Sherbrooke, Canada; 2 Unité de Santé Internationale, Université de Montréal, Canada; 3 Department of Microbiology and Infectious Diseases, Hôpital Maisonneuve-Rosemont, Montréal, Canada; 4 Centre Hospitalier Universitaire de Sherbrooke, Canada; University of Toronto, Canada

## Abstract

**Background:**

The spectrum of bacteria associated with bacterial vaginosis (BV) has recently expanded through taxonomic changes and the use of molecular methods. These methods have yet to be used in large-scale epidemiological studies in Africa where BV is highly prevalent.

**Methods:**

An analysis of samples obtained during a clinical trial of the management of vaginal discharge in four West African countries. Samples were available from 1555 participants; 843 (54%) had BV. Nucleic acids of 13 bacterial genera or species potentially associated with BV were detected through the polymerase chain reaction.

**Results:**

The associations between various components of the vaginal flora were complex. Excluding *Lactobacillus*, the other 12 micro-organisms were all associated with each other at the p≤0.001 level. The prevalence of various bacterial genera or species varied according to age, sexual activity and HIV status. In multivariate analysis, the presence of *Gardnerella vaginalis*, *Bifidobacterium*, *Megasphaera elsdenii*, *Dialister*, *Mycoplasma hominis*, *Leptotrichia*, and *Prevotella* were independently associated with BV as was the absence of *Lactobacillus* and *Peptoniphilus*. However, *Mobiluncus*, *Atopobium vaginae*, *Anaerococcus*, and *Eggerthella* were not independently associated with BV. Unexpectedly, after treatment with a regimen that included either metronidazole or tinidazole, the proportion of patients with a complete resolution of symptoms by day 14 increased with the number of bacterial genera or species present at enrolment.

**Conclusions:**

Numerous bacterial genera or species were strongly associated with each other in a pattern that suggested a symbiotic relationship. BV cases with a simpler flora were less likely to respond to treatment. Overall, the vaginal flora of West African women with BV was reminiscent of that of their counterparts in industrialized countries.

## Introduction

Bacterial vaginosis (BV) is the most common cause of vaginal discharge, both in industrialized and developing countries and among the HIV-infected and uninfected [1], [2]. Its main detrimental effect on pregnancy is preterm delivery [3]. Cross-sectional and cohort studies have revealed a bidirectional association between BV and HIV infection [4]–[7]. Meta-analyses estimated that BV increases the risk of male-to-female transmission of HIV by 40–60% [8]. To date, there is no evidence that treatment of BV reduces the risk of HIV, but the high prevalence of BV suggests that its population-attributable fraction of incident HIV among women could be substantial. By increasing genital shedding of HIV, BV might also impact on female-to-male HIV transmission [9].

BV is attributed to a disturbance in the vaginal flora, with fewer lactobacilli and increasing numbers of anaerobic Gram-negative rods. Its etiological agents remain debated, as BV appears to be a polymicrobial process with interrelated organisms leading to a common outcome. *Gardnerella vaginalis* is only one among several bacterial genera or species that are more common or present in larger quantities in women with BV compared to healthy controls; others include *Mycoplasma hominis*, *Mobiluncus* spp., *Prevotella* spp., *Atopobium vaginae*, *Eggerthella* spp., *Megasphaera* spp., *Leptotrichia* spp., *Peptoniphilus* spp., *Anaerococcus* spp., *Dialister* spp. and novel bacteria in the order *Clostridiales* [10]–[17].

Studies on the microbial correlates of BV have been undertaken in industrialized countries, but less is known about the association between these bacteria and BV in Sub-Saharan Africa, where BV is extremely common and could impact on HIV transmission [18]–[20]. Furthermore, no study using nucleic acid amplification measured the association between multiple genera or species and BV in a population large enough for the confounding effects of multiple organisms to be taken into consideration. To better understand the microbiology of BV in Africa and ultimately to develop more effective treatments, we looked for putative pathogens among participants in a study of the vaginal discharge syndrome in West Africa.

## Methods

This study is a sub-analysis of data collected during a randomized controlled trial for the management of symptomatic vaginal discharge. Subjects presenting with symptoms of vaginal discharge were randomized to metronidazole 500 mg twice a day for seven days plus clotrimazole cream for three days versus single-dose treatment with tinidazole (2 g) plus fluconazole (150 mg) (ClinicalTrials.gov NCT00313131) [2].

### Ethics statement

The protocol was approved by the Ethical Review Committee of the Ghana Health Service, the *Comité National d'Éthique pour la Recherche en Santé* (Guinée), the *Comité d'Éthique de l'Institut National de Recherche en Santé Publique du Mali*, the *Comité d'Éthique sur la Recherche en Santé* (Togo), and the *Comité d'Éthique de la Recherche sur l'Humain du Centre Hospitalier Universitaire de Sherbrooke et de la Faculté de Médecine de l'Université de Sherbrooke* (Canada).

### Data collection

Between January 2004 and April 2005, women complaining of vaginal discharge were recruited in nine healthcare facilities in four West African countries: i) in Ghana, the sexually transmitted infections (STI) clinics of Accra-Adabraka and Kumasi-Suntreso; ii) in Togo, the STI clinics of Amoutivé, Agoé-Nyivé and Adakpamé; iii) in Conakry, Guinea, the Madina, and Carrière health centers; iv) in Bamako, Mali, the Korofina, and Soutoura health centers. Pregnant women, those who complained of abdominal pain, those who were not local residents, and those with allergies to one or more study drugs were excluded. After giving written informed consent, participants were identified only by number. Laboratory assays were performed anonymously through an unlinked method. Participants wishing to know their HIV status received pre-test counseling and a duly identified sample was obtained. Processing of this sample, post-test counseling and referral to a treatment facility were performed per clinic routine.

At the initial visit, a questionnaire gathered demographic, behavioral, and clinical information. Samples were obtained from vaginal and cervical secretions. First, a vaginal fluid sample was deposited in a transport medium and used for the detection of pathogens by the polymerase chain reaction (PCR). A second vaginal sample was used to deposit secretions on a slide that was Gram stained to detect the presence of BV, defined as a Nugent score ≥7 [21]. Yeast, with phenotype compatible with *Candida albicans*, was noted when present. A cervical sample was tested by PCR for the presence of *Neisseria gonorrhoeae*, *Chlamydia trachomatis*, and *Mycoplasma genitalium*. HIV serology was performed on capillary blood [2]. All enrolled participants were randomized to one of the treatment regimens and were asked to return 14 days later to document their symptomatic response.

### Detection of vaginal pathogens

Methods for PCR assays are described in Text S1 and Table S1 [14], [22]–[27]. Initially, we sought to detect nucleic acids of *Trichomonas vaginalis*, *G. vaginalis*, *A. vaginae*, *Prevotella* spp., *Mobiluncus* spp., *M. hominis* and *Lactobacillus* spp in all vaginal samples obtained upon enrolment. During the course of the study, as the number of putative pathogens reported by other researchers increased, a preliminary screening of additional bacterial genera or species was performed on randomly selected samples with (n = 100) and without BV (n = 100). Testing of all samples was performed only for pathogens that showed an association with BV at the p<0.05 level on this subset, including *Leptotrichia* spp., *Eggerthella* spp., *Megasphaera elsdenii*, *Dialister* spp., *Bifidobacterium* spp., *Peptoniphilus* spp. with primers that excluded *P. lacrimalis*, and *Anaerococcus* spp. BVAB-1, BVAB-2, BVAB-3, *Peptoniphilus lacrimalis*, and the *Clostridium coccoides* group were not associated with BV at the p<0.05 level, and therefore, all samples were not tested for these species.

### Data analysis

Data were analyzed with Stata 10.0 (StataCorp LP, College Station, Texas). Proportions were compared with the χ^2^ test. In preliminary analyses, we first examined the associations between various bacterial genera or species. We also evaluated the distribution of bacterial species or genera according to age, HIV status, and number of sexual partners. In the main analysis, we examined the association between various bacterial genera or species and the outcome, which was BV defined as per a Nugent score ≥7. Logistic regression was used for multivariate analysis. Models were built sequentially starting with the variable most strongly associated with the outcome and continuing until no other variable reached significance or altered the odds ratios of variables already in the model. When the final model was reached, each variable was dropped in turn to assess its effect using the likelihood ratio test. Results are presented as adjusted odds ratios (AOR) with their 95% confidence intervals (CI).

## Results

Samples were available from 1555 participants, of whom 843 (54%) had BV. As reported elsewhere, 30% had candidiasis, 10% had trichomoniasis, and cervical pathogens (*N. gonorrhoeae*, *C. trachomatis*, or *M. genitalium*) were found in 11%, while 32% of cases had no clear etiology of the vaginal discharge elicited [2]. Table 1 shows the associations between microorganisms among all participants as correlation coefficients, while Table S2 displays the distribution of bacterial species or genera. *Lactobacillus* was the most frequently detected, followed by *Bifidobacterium*. Conversely, *Mobiluncus* was rarely detected. Excluding *Lactobacillus*, the other 12 micro-organisms were all associated with each other at the p≤0.001 level. In contrast, *Lactobacillus* poorly correlated with the presence or absence of other microorganisms, and those associations that were significant because of the large sample size were not impressive for their absolute differences in prevalence.

**Table 1:** Associations between various bacterial species and genera.^1^

	GV	MH	AV	MO	PR	AN	BI	DI	LE	ME	PE	LA
**GV**	1	0.320	0.580	0.085	0.394	0.288	0.517	0.304	0.367	0.181	0.206	0.112
**MH**		1	0.365	0.168	0.331	0.228	0.256	0.339	0.421	0.159	0.195	0.020
**AV**			1	0.139	0.384	0.417	0.395	0.375	0.388	0.250	0.250	0.084
**MO**				1	0.207	0.135	0.091	0.221	0.215	0.106	0.107	−0.136
**PR**					1	0.286	0.378	0.401	0.464	0.181	0.275	−0.042
**AN**						1	0.327	0.432	0.343	0.222	0.306	−0.054
**BI**							1	0.397	0.400	0.216	0.236	−0.097
**DI**								1	0.510	0.261	0.400	−0.046
**LE**									1	0.391	0.245	−0.093
**ME**										1	0.086	−0.079
**PE**											1	0.083
**LA**												1

1 Correlation coefficients (*phi*) were used to measure associations between dichotomic variables and are displayed in the table. All correlations had p-values <0.001, excepted LA-ME (0.002), LA-AN (0.03), LA-DI (0.07), LA-PR (0.10), and LA-MH (0.43).

Note: GV: *Gardnerella vaginalis*; AV: *Atopobium vaginae*; MH: *Mycoplasma hominis*;

PR: *Prevotella*; MO: *Mobiluncus*; LE: *Leptotrichia*; ME: *Megasphaera elsdenii*;

EG: *Eggerthella*; DI: *Dialister* (DI); BI *Bifidobacterium* (BI); AN: *Anaerococcus*; PE *Peptoniphilus* other than *lacrimalis*; LA: *Lactobacillus* (LA).

We examined the distribution of various bacterial genera or species among all participants. To simplify the presentation and comparisons between microorganisms, Table 2 displays the results of a common multivariate model, using each micro-organism as the outcome sequentially. More detailed data are available in Tables S3, S4, S5, and S6. In the West African societies where this recruitment occurred, most women (440/472 [93%]) who reported having had more than one sexual partner over the preceding three months were sex workers with numerous partners. Older age was associated with lower likelihood of colonization/infection with *G. vaginalis*, *M. hominis*, *A. vaginae*, *Eggerthella*, *Leptotrichia*, *Dialister*, and *Bifidobacterium*. Being sexually active was associated with colonization/infection with *G. vaginalis*, *Prevotella*, *Mobiluncus*, *Eggerthella*, *Leptotrichia*, and *Bifidobacterium* and was inversely associated with *Lactobacillus* colonization. For some pathogens, there was little difference related to the degree of sexual activity (comparing ≥2 partners to 1 partner), while others (*Prevotella*, *Eggerthella*, *Leptotrichia* and *Bifidobacterium*) were more prevalent among women with ≥2 partners. HIV infection was associated with colonization/infection with *G. vaginalis*, *M. hominis*, *Prevotella*, *Mobiluncus*, *Eggerthella*, *Leptotrichia*, *Dialister*, *Bifidobacterium*, and *Peptoniphilus* non-*lacrimalis*, while it decreased the likelihood of being colonized with *Lactobacillus*.

**Table 2:** Correlates of colonization/infection with various bacterial genera or species.

	*G. vaginalis*	*M. hominis*	*A. vaginae*	*Prevotella*	*Mobiluncus*
**Age, years**					
≤20	1.00	1.00	1.00	1.00	1.00
21–30	0.82 (0.64–1.06)	0.67 (0.50–0.90)^2^	0.77 (0.60–0.99)^2^	0.98 (0.76–1.27)	1.52 (0.83–2.77)
≥31	0.59 (0.44–0.81)^1^	0.46 (0.32–0.67)^1^	0.73 (0.54–0.99)^2^	0.91 (0.67–1.24)	1.14 (0.56–2.35)
**Sex partners, last 3 months**					
None	1.00	1.00	1.00	1.00	1.00
One	1.37 (0.98–1.91)	1.27 (0.84–1.92)	1.20 (0.86–1.70)	1.04 (0.74–1.46)	3.02(1.08–8.47)^2^
Two or more	1.45 (1.01–2.07)^2^	1.14 (0.73–1.76)	1.16 (0.81–1.67)	1.58 (1.10–2.26)^2^	1.73 (0.58–5.15)
**HIV**					
Negative	1.00	1.00	1.00	1.00	1.00
Positive	1.77 (1.29–2.42)^1^	2.06 (1.47–2.87)^1^	1.12 (0.82–1.52)	1.86 (1.37–2.54)^1^	2.19(1.27–3.80)^2^

	*Eggerthella*	*M. elsdenii*	*Leptotrichia*	*Dialister*	*Bifidobacterium*
**Age, years**					
≤20	1.00	1.00	1.00	1.00	1.00
21–30	0.73 (0.56–0.95)^2^	0.94 (0.66–1.33)	0.71 (0.55–0.92)^2^	0.78 (0.59–1.04)	0.74(0.56–0.96)^2^
≥31	0.48 (0.34–0.68)^1^	0.81 (0.52–1.25)	0.53 (0.38–0.73)^1^	0.60 (0.42–0.86)^2^	0.50(0.36–0.68)^1^
**Sex partners, last 3 months**					
None	1.00	1.00	1.00	1.00	1.00
One	1.29 (0.87–1.91)	1.32 (0.80–2.17)	1.26 (0.88–1.81)	1.10 (0.74–1.64)	1.29 (0.92–1.81)
Two or more	1.59 (1.05–2.40)^2^	1.21 (0.71–2.06)	1.81 (1.24–2.66)^2^	1.26 (0.83–1.91)	1.61 (1.11–2.32)^2^
**HIV**					
Negative	1.00	1.00	1.00	1.00	1.00
Positive	1.41 (1.02–1.96)^2^	1.03 (0.67–1.57)	1.59 (1.17–2.17)^2^	1.73 (1.25–2.40)^1^	2.07(1.46–2.93)^1^

	*Anaerococcus*	*Peptoniphilus* 3	*Lactobacillus*
**Age, years**			
≤20	1.00	1.00	1.00
21–30	0.88 (0.65–1.20)	0.93 (0.68–1.27)	1.07 (0.79–1.44)
≥31	0.76 (0.51–1.11)	0.68 (0.46–1.01)	0.99 (0.70–1.42)
**Sex partners, last 3 months**			
None	1.00	1.00	1.00
One	1.06 (0.70–1.62)	0.80 (0.52–1.21)	0.58 (0.37–0.90)^2^
Two or more	1.01 (0.65–1.58)	1.13 (0.73–1.75)	0.54 (0.34–0.87)^2^
**HIV**			
Negative	1.00	1.00	1.00
Positive	1.13 (0.78–1.63)	1.88 (1.34–2.65)^1^	0.66 (0.47–0.92)^2^

1 ≤0.001.

2 ≤0.05.

3 other than *lacrimalis*.

Results presented are the adjusted odds ratios with their 95% confidence intervals. More detailed data are available in Tables S3, S4, S5, and S6.

Table 3 shows the correlates of BV in univariate analyses. BV was more frequent in younger women, sexually active women, and HIV-infected women. BV prevalence was equal among sex workers and non-sex workers. BV was strongly associated with *G. vaginalis*, *M. hominis*, *A. vaginae*, *Prevotella*, *Mobiluncus*, *Leptotrichia*, *M. elsdenii*, *Eggerthella*, *Dialister*, *Bifidobacterium*, and *Anaerococcus* (crude odds ratios ≥3.0) and less strongly associated with *Peptoniphilus* non-*lacrimalis* and pathogens not thought to be causal agents of BV (*T. vaginalis*, *C. trachomatis*, or *M. genitalium*) (crude odds ratios ≤2.0). Presence of *Lactobacillus* was associated with lower odds of BV. Yeast infections were equally frequent in women with or without BV.

**Table 3:** Correlates of bacterial vaginosis in univariate analyses.

	Bacterial vaginosis (%)	No bacterial vaginosis (%)	Odds ratio
	(Nugent score≥7)	(Nugent score<7)	(95% CI)
**Age, years**			
≤20	206 (52)	194 (48)	1.00
21–30	354 (45)	427 (55)	0.79 (0.62–1.00)
≥31	149 (41)	212 (59)	0.68 (0.51–0.90)^2^
**Sex worker**			
No	446 (46)	523 (54)	1.00
Yes	263 (46)	310 (54)	0.99 (0.81–1.22)
**Sex partners, last 3 months**			
None	68 (38)	112 (62)	1.00
One	428 (48)	456 (52)	1.58 (1.14–2.20)^2^
Two or more	212 (45)	260 (55)	1.36 (0.96–1.93)
**HIV**			
Negative	546 (44)	690 (56)	1.00
Positive	119 (55)	97 (45)	1.56 (1.17–2.07)^2^
***Bifidobacterium***			
Negative	120 (21)	454 (79)	1.00
Positive	595 (61)	379 (39)	5.94 (4.68–7.54)^1^
***Leptotrichia***			
Negative	302 (31)	673 (69)	1.00
Positive	411 (72)	161 (28)	5.69 (4.53–7.14)^1^
***Dialister***			
Negative	423 (36)	738 (64)	1.00
Positive	292 (75)	95 (25)	5.36 (4.13–6.96)^1^
***Eggerthella***			
Negative	397 (36)	718 (64)	1.00
Positive	318 (73)	116 (27)	4.96 (3.88–6.34)^1^
***Megasphaera elsdenii***			
Negative	537 (41)	781 (59)	1.00
Positive	178 (77)	53 (23)	4.88 (3.53–6.77)^1^
***Mycoplasma hominis***			
Negative	456 (38)	748 (62)	1.00
Positive	259 (74)	92 (26)	4.62 (3.54–6.02)^1^
***Mobiluncus***			
Negative	648 (44)	820 (56)	1.00
Positive	66 (78)	19 (22)	4.40 (2.61–7.40)^1^
***Gardenerella vaginalis***			
Negative	195 (27)	517 (73)	1.00
Positive	520 (62)	323 (38)	4.27 (3.44–5.29)^1^
***Atopobium vaginae***			
Negative	309 (33)	626 (67)	1.00
Positive	406 (65)	214 (35)	3.84 (3.10–4.76)^1^
***Prevotella***			
Negative	258 (31)	573 (69)	1.00
Positive	456 (63)	266 (37)	3.81 (3.08–4.70)^1^
***Anaerococcus***			
Negative	494 (40)	744 (60)	1.00
Positive	220 (71)	89 (29)	3.72 (2.84–4.88)^1^
***Peptoniphilus*** ** other than ** ***lacrimalis***			
Negative	544 (44)	695 (56)	1.00
Positive	170 (55)	138 (45)	1.58 (1.22–2.02)^1^
***Lactobacillus***			
Negative	239 (66)	122 (34)	1.00
Positive	476 (40)	718 (60)	0.34 (0.26–0.43)^1^
***Trichomonas vaginals***			
Negative	622 (44)	778 (56)	1.00
Positive	93 (60)	62 (40)	1.94 (1.39–2.72)^1^
***Neisseria gonorrhoeae***			
Negative	676 (45)	810 (55)	1.00
Positive	39 (57)	30 (42)	1.52 (0.94–2.48)
***Chlamydia trachomatis***			
Negative	678 (45)	817 (55)	1.00
Positive	37 (62)	23 (38)	2.00 (1.19–3.39)^2^
***Mycoplasma genitalium***			
Negative	667 (45)	811 (55)	1.00
Positive	48 (62)	29 (38)	2.06 (1.29–3.28)^2^
**Yeasts**			
Negative	507 (47)	576 (53)	1.00
Positive	208 (44)	264 (56)	0.89 (0.71–1.10)

1 ≤0.001.

2 ≤0.05.

Table 4 shows the results of the multivariate analysis of correlates of BV. Number of sexual partners increased the fit of the model even if each category was not significant. Age and HIV status became non-significant and were left out. The presence of *G. vaginalis*, *Bifidobacterium*, *M. elsdenii*, *Dialister*, *M. hominis*, *Leptotrichia*, and *Prevotella* was independently associated with BV as was the absence of *Lactobacillus* and *Peptoniphilus* non-*lacrimalis*. When fitted into this model, the following pathogens were no longer independently associated with BV: *A. vaginae* (AOR: 1.30; 95% CI: 0.95–1.78; p = 0.10), *Mobiluncus* (AOR: 1.36; 95% CI: 0.72–2.56; p = 0.34), *Anaerococcus* (AOR: 1.17; 95% CI: 0.83–1.66; p = 0.37) and *Eggerthella* (AOR: 1.03; 95% CI: 0.71–1.50; p = 0.86). The presence of *T. vaginalis*, *N. gonorrhoeae*, *C. trachomatis*, or *M. genitalium* was not independently associated with BV after adjustment for the other correlates of BV (data not shown).

**Table 4:** Correlates of bacterial vaginosis (Nugent score ≥7) in multivariate analysis.

	Adjusted odds ratio (95% CI)
**Sex partners, last 3 months**	
None	1.00
One	1.39 (0.92–2.08)
Two or more	0.85 (0.55–1.31)
***Gardenerella vaginalis***	
Negative	1.00
Positive	2.35 (1.74–3.16)^1^
***Bifidobacterium***	
Negative	1.00
Positive	2.22 (1.64–3.01)^1^
***Megasphaera elsdenii***	
Negative	1.00
Positive	2.09 (1.41–3.10)^1^
***Dialister***	
Negative	1.00
Positive	1.99 (1.39–2.83)^1^
***Mycoplasma hominis***	
Negative	1.00
Positive	1.91 (1.39–2.64)^1^
***Leptotrichia***	
Negative	1.00
Positive	1.72 (1.26–2.36)^1^
***Prevotella***	
Negative	1.00
Positive	1.53 (1.15–2.03)^2^
***Peptoniphilus*** ** other than ** ***lacrimalis***	
Negative	1.00
Positive	0.56 (0.40–0.79)^1^
***Lactobacillus***	
Negative	1.00
Positive	0.26 (0.19–0.35)^1^

1 ≤0.001.

2 ≤0.05.

The robustness of our conclusions was tested on alternative models. As it could be argued that the presence of *Lactobacillus* by PCR was too directly related to the Gram stain definition of BV, this variable was excluded in a subsequent model, which showed that the same eight micro-organisms were associated with BV, while the same four were not significantly associated (data not shown). In models (with or without *Lactobacillus*) that excluded women with an intermediate Nugent score (4–6), *G. vaginalis*, *Bifidobacterium*, *Dialister*, *M. hominis*, *Leptotrichia*, and *Prevotella* remained associated with BV, while the presence of *M. elsdenii* and the absence of *Peptoniphilus* non-*lacrimalis* became non-significant (Tables S7 and S8). Likewise, *A. vaginae*, *Mobiluncus*, *Eggerthella*, and *Anaerococcus* were not significantly associated with BV.

Of 349 women without evidence of *G. vaginalis*, *M. hominis*, *Prevotella*, *Leptotrichia*, *M. elsdenii*, *Dialister*, and *Bifidobacterium*, only 12% (41) had BV as diagnosed by Nugent score. This proportion increased to 25% (54/220), 44% (104/236), 53% (101/189), 68% (126/184), 70% (120/171), 84% (133/158) and 89% (33/37) for women with respectively one, two, three, four, five, six or all of these microorganisms respectively (p<0.001, χ^2^ for trend).

In a secondary analysis that included all 570 women with BV who attended their follow-up visit on day 14 (Table S9), the proportion with a complete resolution of symptoms increased with the number of pathogens identified on day 0: 41% of those with a single pathogen, and 57%, 56%, 58%, 70%, 64%, 70%, and 76% of those with one, two, three, four, five, six, or seven pathogens respectively (p<0.001, χ^2^ for trend) had complete resolution of symptoms. This trend was stronger in participants who had been treated with single-dose tinidazole/fluconazole than for those treated with metronidazole/clotrimazole. Age and HIV status had no impact on the likelihood of a complete short-term response to treatment. In a multivariate analysis, complete resolution of symptoms on day 14 was more common in women in whose samples *Prevotella* (AOR: 1.91; 95% CI: 1.34–2.74; p<0.001) or *M. elsdenii* (AOR: 1.54; 95% CI: 1.01–2.34; p = 0.04) had been detected and among those with ≥2 sex partners (AOR: 1.95; 95% CI: 0.97–3.93; p = 0.06). No other pathogens or the treatment allocation was independently associated with this complete short-term resolution of symptoms.

## Discussion

We documented complex interrelationships between several micro-organisms present in the vaginal flora of a large number of women presenting with vaginal discharge in West Africa. These organisms were strongly associated with each other to an extent that can hardly be explained by shared risk factors for transmission but likely indicates a symbiotic relationship within the vaginal ecosystem [10], [11]. Many organisms were associated with BV in univariate analyses but four became non-significant after adjustment for the presence of other bacteria. Previous analyses of the microbiology of BV considered only some of these organisms, used only univariate analyses, or combined all pathogens into a composite variable [11]–[15], [17]. Our sample size was substantially larger than all previous studies and allowed us to adjust for the concomitant presence of multiple bacteria. Amongst our participants, *Mobiluncus*, *Eggerthella*, *Anaerococcus*, and *A. vaginae* were not independently associated with BV but were rather confounded by their association with other genera or species that themselves were correlates of BV. Our results support the idea that *G. vaginalis*, *Bifidobacterium*, *M. elsdenii*, *Dialister*, *M. hominis*, *Leptotrichia*, and *Prevotella* are associated with BV and that its microbiology is the same in Africa as in industrialized countries.

Our interpretation of Table 2 is as follows. Colonization/infection with several organisms was enhanced by sexual activity and by HIV-associated immunosuppression. Some organisms must be transmitted during intercourse. For others, organisms that are equally prevalent in sex workers and monogamous sexually active women, a non-specific effect of intercourse on the vaginal flora seems plausible and may support a recent description of BV as a “sexually enhanced disease” [28]. For most organisms, prevalence decreased with age, perhaps through a decrease in sexual activity or the progressive development of mucosal immunity, which may wane with HIV infection. The relative protection against BV conferred by age and the higher risk with sexual activity and HIV infection are consistent with findings for some of the organisms individually. In multivariate analysis, HIV and younger age were no longer risk factors for BV, because they were associated with colonization/infection with several bacterial genera or species whose inclusion in the model removed the effect of HIV and age, which lay further away in the causal pathway.

Metronidazole is an imperfect treatment of BV: 10–20% of patients do not respond, 15–30% relapse within 3 months and half relapse within one year [1], [29]. Whether relapses may be due to re-infections accompanying intercourse remains controversial [29], [30]. Systematically administered metronidazole has little or no activity against *M. hominis*, *Bifidobacterium*, and *G. vaginalis* and only moderate activity against *Dialister* [31], [32]. Metronidazole has predictable activity against *Prevotella*, *Leptotrichia*, and *Eggerthella* [33], [34]. Despite this resistance, metronidazole treatment does reduce vaginal counts of *M. hominis*, *G. vaginalis*, *Prevotella*, *Megasphaera*, and to a lower extent *Leptotrichia*, with little difference between oral or intravaginal administration [32], [35]–[37]. Topical clindamycin, the other recommended treatment for BV [38], is not ideal either as one-sixth of vaginal anaerobes at baseline are resistant, and clindamycin resistance can emerge during treatment, especially in *Prevotella* [32], [39]. Nevertheless, clindamycin appears equivalent to metronidazole in achieving a clinical cure [40].

Counter-intuitively, we showed that after a regimen that included either metronidazole or tinidazole, the proportion of patients with a complete resolution of symptoms by day 14 increased with the number of pathogens present on day 0 and that the presence of *Prevotella* was strongly associated with a good short-term response. These findings may reflect the symbiotic nature of BV such that the reduction of the metronidazole-susceptible portion of BV pathogens results in a lack of sustainability of the more resistant organisms and a net overall effect of clinical cure. In the presence of multiple pathogens, the *G. vaginalis*-related biofilm may be less important in the pathogenesis of BV [41].

Our study had limitations. First, we had to be selective in the choice of pathogens to be tested on all samples, for financial and logistical reasons. Additional BV correlates (for instance, the BV-associated bacteria in the *Clostridiales* order, other *Megasphaera* species, *Papillibacter*, or *Lachnospiraceae*) may have been identified had we not faced this restriction [20], [42], [43]. Second, the recruitment was performed in West Africa; whether the vaginal flora varies between geographic regions is unknown but it does seem to vary according to ethnicity, and genetically determined factors could be involved in the pathogenesis of BV, as suggested by studies in the United States where the disease is more prevalent among African-American women, even after adjustment for sexual behavior and other confounders [44], [45]. Third, all participants, including those that became the control group, had consulted for vaginal discharge. One third of participants without BV had yeast visible on Gram stain, but there is so far little evidence that the bacterial flora of women with vaginal candidiasis is altered as compared to that of healthy women [46]. Fourth, using extremely sensitive molecular methods, we looked for pathogens whose presence or absence contributed, to various extents, to the definition of the outcome through the Nugent score. This problem was potentially worse for *Lactobacillus*, whose morphotype (large Gram-positive rods) accounts for 4/10 points. However, inclusion or exclusion of *Lactobacillus* positivity by PCR had no effect on the association of other pathogens with BV. Furthermore, *Mobiluncus*, a curved Gram-variable rod whose morphotype corresponds to two points, was not associated independently with the outcome. Presumably, the overlap in the Gram stain morphology between the various constituents of the vaginal flora and the large number of organisms that we tested by PCR enabled us to avoid this circular reasoning.

In conclusion, among West African women with BV, numerous bacterial micro-organisms were strongly associated with each other in a pattern that suggested a symbiotic relationship. Overall, the vaginal flora of West African women with BV was reminiscent of that of their counterparts in industrialized countries. Cases of BV with a simpler flora were less likely to respond to metronidazole or tinidazole. A better understanding of the determinants of therapeutic response is needed before more effective treatments can be developed.

## Supporting Information

Table S1 **Primers used for various pathogens.**
(DOC)

Table S2 **Frequency of associations between various bacterial species or genera.**
(DOC)

Table S3 **Prevalence of micro-organisms according to age.**
(DOC)

Table S4 **Prevalence of micro-organisms according to self-defined occupation.**
(DOC)

Table S5 **Prevalence of micro-organisms according to number of sex partners in the last 3 months.**
(DOC)

Table S6 **Prevalence of micro-organisms according to HIV status.**
(DOC)

Table S7 **Correlates of bacterial vaginosis (Nugent score ≥7) in multivariate analysis, excluding patients with an intermediate Nugent score (4–6).**
(DOC)

Table S8 **Correlates of bacterial vaginosis (Nugent score ≥7) in multivariate analysis, excluding patients with an intermediate Nugent score (4–6). Presence of Lactobacillus has been removed from this model.**
(DOC)

Table S9 **Proportion of patients with bacterial vaginosis who reported a complete resolution of vaginal discharge by Day 14.**
(DOC)

Text S1 **Methods used for nucleic acid amplification testing for various bacterial genus or species.**
(DOC)
