# Supplementary material for: The Complex Vaginal Flora of West African Women with Bacterial Vaginosis
Source: PLoS One. 2011 Sep 20;6(9):e25082. doi: 10.1371/journal.pone.0025082 (PMC3176826; doi:10.1371/journal.pone.0025082)
Supplement: Table S1 — Primers used for various pathogens. (DOC) [file pone.0025082.s001.doc]

**Table S1. Primers used for various pathogens.**

| Target | Sequences | Size | Reference |
| --- | --- | --- | --- |
| *G. vaginalis* (23S rRNA gene) | | | |
| Forward primer Gard382F | TTACTGGTGTATCACTGTAAGG | 334 bp | 24 |
| Reverse primer Gard714R | CCGTCACAGGCTGAACAGT |  | 24 |
| *A. vaginae* (16S rRNA gene) | | | |
| Forward primer Ato167F | GCGAATATGGGAAAGCTCCG | 533 bp | 22 |
| Reverse primer Ato715R | TCATGGCCCAGAAGACCGCC |  | 25 |
| *Lactobacillus* sp (16S rRNA gene) | | | |
| Forward primer Lac739F | GCGGCTCTCTGGTCTGCAAC | 128 bp | This report |
| Reverse primer Lac866R | AGCACTGAGAGGCGGAAACCT |  | This report |
| *M. hominis* (16S rRNA gene) | | | |
| Forward primer MH141F | CAATGGCTAATGCCGGATACGC | 334 bp | 26 |
| Reverse primer MH475R2 | GGTACCGTCAGTCTGCAATCATT |  | This report |
| *Prevotella* sp. (16S rRNA gene) | | | |
| Forward primer PrevRNA810F | CACRgTAAACgATggATgCC | 514 bp | 23 |
| Reverse primer  PrevRNA1322R | ggTCgggTTgCAgACC |  | 23 |
| *Mobiluncus* sp. (16S rRNA gene) | | | |
| Forward primer MobRNA397F | ggTTGTgAACTCCTTTTTCTCgYgAA | 427 bp | 27 |
| Reverse primer MobRNA823R | CgCAgAAACACAggATAgCATCC |  | This report |
| *Leptotrichia* sp (16S rRNA gene) | | | |
| Forward primer Lepto395F | CAATTCTGTGTGTGTGAAGAAG | 252 bp | 14 |
| Reverse primer  Lepto646R | ACAGTTTTGTAGGCAAGCCTAT |  | 14 |
| Probe  Lepto-605R665 | TYE665 - CACCTTCAACTTGTCTTACCGCCT AGATG – IBRQ |  |  |
| *M. elsdenii* (16S rRNA gene) | | | |
| Forward primer MegaE456F | GATGCCAACAGTATCCGTCCG | 212 bp | 14 |
| Reverse primer MegaE-667R | CCTCTCCGACACTCAAGTTCGA |  | 14 |
| Probe  MegaE-616R615 | TEX615 - CCGCACTTTTAAGACAGACTTACC GAAC_- IBRQ |  | This report |
| *Eggerthella* sp. (16S rRNA gene) | | | |
| Forward primer Egger621F | AACCTCGAGCCGGGTTCC | 239 bp | 14 |
| Reverse primer Egger859R | TCGGCACGGAAGATGTAATCT |  | 14 |
| Probe  Egger838R-Cyan | CYAN500-CCACACCCAGCGCTCATCGTTTACGG—BBQ |  | This report |
| *Dialister* sp. (16S rRNA gene) | | | |
| Forward primer Dial476F | TGACGGTACCGGAAAAGC | 334 bp | This report |
| Reverse primer Dial662R | CTCTCCGATACTCCAGCTTC |  | This report |
| Probe  Dialis601F563 | TYE563 – TCCATCTTAAAAGCGTGGGGCTCA AC – IBRQ |  | This report |
| *Bifidobacterium* sp. (16S rRNA gene) | | | |
| Forward primer Bifi592F | CTCGTCGCGTCYGGTGTGA | 514 bp | 23 |
| Reverse primer  Bifi836R | CCACATCCAGCRTCCAC |  | 23 |
| Probe  Bifido689F665 | TYE665 – AACGGTGGAATGTGTAGATATCGG GAA – IBRQ |  | This report |
| *Anaerococcus* sp. (16S rRNA gene) | | | |
| Forward primer AnCoc318F | ATTGGGACTGAGACACGGC | 334 bp | This report |
| Reverse primer AnCoc642R | CACTAGGAATTCCACTTTCCCT |  | This report |
| Probe  AncocV556FFam | FAM – ATCATTGGGCGTAAAGGGTACGTAG – IBFQ |  | This report |
| *Peptoniphilus* other than *lacrimalis* (16S rRNA gene) | | | |
| Forward primer Pepton1003F | GACCGGTATAGAGATATACCCT | 182 bp | 14 |
| Reverse primer Pepton1184R | CACCTTCCTCCGATTTATCATC |  | 14 |
| Probe  PepSP1134R563 | TYE563 – CCCATCCGAAATGCTGGTAACTAA AGAT – IBRQ |  | This report |
| *Peptoniphilus lacrimalis* (16S rRNA gene) | | | |
| Forward primer P.lacri-999F | AAGAGACGAACTTAGAGATAAGTTTT | 186 bp | 14 |
| Reverse primer same as Pepton1184R | CACCTTCCTCGATTTATCATC |  | 14 |
| BVAB-1 (16S rRNA gene) | | | |
| Forward primer BVAB1-1019F | GTATATTTTCTACGGAACACAGG | 262 bp | 14 |
| Reverse primer  BVAB1-1280R | TTTGCTCCGGATCGCTCCTT | 186 bp | 14 |
| BVAB-2 (16S rRNA gene) | | | |
| Forward primer BVAB2-619F | TTAACCTTGGGGTTCATTACAA | 406 bp | 14 |
| Reverse primer BVAB2-1024R | AATTCAGTCTCCTGAATCGTCAGA | 262 bp | 14 |
| BVAB-3 (16S rRNA gene) | | | |
| Forward primer BVAB3-999F | CTTGAWCGATGTAGAGATACATAA | 334 bp | 14 |
| Reverse primer BVAB3-1278R | TGCTTCGCCTCGCGACGTC | 406 bp | 14 |
| *Clostridium coccoides* group (16S rRNA gene) | | | |
| Forward primer Ccoc468F | CGGTACCTGACTAAGAAGC | 334 bp | Modified from 23 |
| Reverse primer Ccoc912R | AGTTTYATTCTTGCGAACG | 334 bp | Modified from 23 |
