# Supplementary material for: The Complex Vaginal Flora of West African Women with Bacterial Vaginosis
Source: PLoS One. 2011 Sep 20;6(9):e25082. doi: 10.1371/journal.pone.0025082 (PMC3176826; doi:10.1371/journal.pone.0025082)
Supplement: Table S2 — Frequency of associations between various bacterial species or genera. (DOC) [file pone.0025082.s002.doc]

**Table S2. Frequency of associations between various bacterial species or genera.**

|  | AV +  (%) | MH +  (%) | PR +  (%) | MO +  (%) | LE +  (%) | ME +  (%) | EG +  (%) | DI +  (%) | BI +  (%) | AN +  (%) | PE +  (%) | LA +  (%) |
| --- | --- | --- | --- | --- | --- | --- | --- | --- | --- | --- | --- | --- |
| GV + (n=843) | 556 (66) | 294 (35) | 543 (65) | 61 (7) | 446 (53) | 175 (21) | 346 (41) | 311 (37) | 720 (86) | 256 (31) | 230 (27) | 684 (81) |
| GV - (n=712) | 64 (9) | 57 (8) | 179 (25) | 24 (3) | 126 (18) | 56 (8) | 88 (12) | 76 (11) | 254 (36) | 53 (7) | 78 (11) | 510 72) |
| AV + (n=620) |  | 256 (41) | 434 (70) | 58 (9) | 371 (60) | 160 (26) | 310 (50) | 278 (45) | 534 (86) | 250 (40) | 199 (32) | 503 (81) |
| AV - (n=935) |  | 95 (10) | 288 (31) | 27 (3) | 201 (22) | 71 (8) | 124 (13) | 109 (12) | 440 (47) | 59 (6) | 109 (12) | 691 (74) |
| MH + (n=351) |  |  | 270 (77) | 44 (13) | 261 (75) | 89 (25) | 218 (62) | 183 (52) | 301 (86) | 129 (37) | 120 (34) | 275 (78) |
| MH - (n=1204) |  |  | 452 (38) | 41 (3) | 311 (26) | 142 (12) | 216 (18) | 204 (17) | 673 (56) | 180 (15) | 188 (16) | 919 (76) |
| PR + (n=722) |  |  |  | 76 (11) | 438 (61) | 157 (22) | 344 (48) | 314 (44) | 593 (82) | 232 (32) | 228 (32) | 541 (75) |
| PR - (n=831) |  |  |  | 9 (1) | 133 (16) | 74 (9) | 90 (11) | 73 (9) | 379 (46) | 77 (9) | 80 (10) | 652 (78) |
| MO + (n=85) |  |  |  |  | 68 (80) | 26 (31) | 58 (68) | 55 (65) | 69 (81) | 36 (42) | 32 (38) | 45 (53) |
| MO - (n=1468) |  |  |  |  | 503 (34) | 205 (14) | 376 (26) | 332 (23) | 903 (62) | 273 (19) | 276 (19) | 1148 (78) |
| LE + (n=572) |  |  |  |  |  | 189 (33) | 383 (67) | 307 (54) | 504 (88) | 216 (38) | 186 (33) | 410 (72) |
| LE - (n=975) |  |  |  |  |  | 41 (4) | 50 (5) | 78 (8) | 468 (48) | 92 (9) | 120 (12) | 778 (80) |
| ME + (n=231) |  |  |  |  |  |  | 163 (71) | 120 (52) | 203 (88) | 95 (41) | 65 (28) | 159 (69) |
| ME - (n=1318) |  |  |  |  |  |  | 271 (21) | 267 (20) | 771 (58) | 214 (16) | 243 (18) | 1030 (78) |
| EG + (n=434) |  |  |  |  |  |  |  | 260 (60) | 392 (90) | 188 (43) | 148 (34) | 309 (71) |
| EG – (n=1115) |  |  |  |  |  |  |  | 127 (11) | 582 (52) | 121 (11) | 160 (14) | 880 (79) |
| DI + (n=387) |  |  |  |  |  |  |  |  | 372 (96) | 193 (50) | 184 (48) | 284 (73) |
| DI - (n=1161) |  |  |  |  |  |  |  |  | 602 (52) | 116 (10) | 124 (11) | 904 (78) |
| BI + (n=974) |  |  |  |  |  |  |  |  |  | 292 (30) | 264 (27) | 717 (74) |
| BI - (n=574) |  |  |  |  |  |  |  |  |  | 17 (3) | 44 (8) | 471 (82) |
| AN+ (n=309) |  |  |  |  |  |  |  |  |  |  | 137 (44) | 223 (72) |
| AN- (n=1238) |  |  |  |  |  |  |  |  |  |  | 171 (14) | 964 (78) |
| PE + (n=308) |  |  |  |  |  |  |  |  |  |  |  | 258 (84) |
| PE - (n=1239) |  |  |  |  |  |  |  |  |  |  |  | 929 (75) |

p ≤0.001 for all comparisons between two pathogens excepted LA-EG (p=0.002), LA-ME (p=0.003), LA-AN (p=0.04), while LA-MH, LA-PR and LA-DI were not significant

Note: GV: *Gardnerella vaginalis*; AV: *Atopobium vaginae*; MH: *Mycoplasma hominis*; PR: *Prevotella*; MO: *Mobiluncus*; LE: *Leptotrichia*; ME: *Megasphaera elsdenii*; EG: *Eggerthella*; DI: *Dialister* (DI); BI *Bifidobacterium* (BI); AN: *Anaerococcus*; PE *Peptoniphilus* other than *lacrimalis*; LA: *Lactobacillus* (LA).
