# Supplementary material for: The Complex Vaginal Flora of West African Women with Bacterial Vaginosis
Source: PLoS One. 2011 Sep 20;6(9):e25082. doi: 10.1371/journal.pone.0025082 (PMC3176826; doi:10.1371/journal.pone.0025082)
Supplement: Table S3 — Prevalence of micro-organisms according to age. (DOC) [file pone.0025082.s003.doc]

**Table S3. Prevalence of micro-organisms according to age.**

|  | Age | | | p-value |
| --- | --- | --- | --- | --- |
|  | 20 years or less | 21-30 years | 31 years or more |  |
| *Gardenerella vaginalis*  Positive/Total | 237/400 (59.3) | 422/781 (54.0) | 176/361 (48.8) | 0.01 |
| *Mycoplasma hominis*  Positive/Total | 114/400 (28.5) | 174/781 (22.3) | 62/361 (17.2) | <0.001 |
| *Atopobium vaginae*  Positive/Total | 179/400 (44.8) | 302/781 (38.7) | 134/361 (37.1) | NS |
| *Prevotella*  Positive/Total | 190/400 (47.5) | 367/779 (47.1) | 161/361 (44.6) | NS |
| *Mobiluncus*  Positive/Total | 15/400 (3.8) | 50/779 (6.4) | 20/361 (5.5) | NS |
| *Eggerthella*  Positive/Total | 139/398 (34.9) | 214/780 (27.4) | 76/358 (21.2) | <0.001 |
| *Megasphaera elsdenii*  Positive/Total | 60/398 (15.1 | 117/780 (15.0) | 49/358 (13.7) | NS |
| *Leptotrichia*  Positive/Total | 174/397 (43.8) | 281/780 (36.0) | 111/357 (31.1) | 0.001 |
| *Dialister*  Positive/Total | 117/398 (29.4) | 189/779 (24.3) | 76/358 (21.2) | 0.03 |
| *Bifidobacterium*  Positive/Total | 272/398 (68.3) | 491/779 (63.0) | 202/358 (56.4) | 0.003 |
| *Anaerococcus*  Positive/Total | 85/398 (21.4) | 153/778 (19.7) | 66/358 (18.4) | NS |
| *Peptoniphilus* other than *lacrimalis*  Positive/Total | 89/398 (22.4) | 157/778 (20.2) | 60/358 (16.8) | NS |
| *Lactobacillus*  Positive/Total | 306/400 (76.5) | 607/781 (77.7) | 271/361 (75.1) | NS |
| *Trichomonas vaginalis*  Positive/Total | 48/400 (12.0) | 73/781 (9.3) | 31/361 (8.6) | NS |
| *Neisseria gonorrhoeae*  Positive/Total | 27/400 (6.8) | 29/781 (3.7) | 11/361 (3.0) | 0.02 |
| *Chlamydia trachomatis*  Positive/Total | 34/400 (8.5) | 19/781 (2.4) | 6/361 (1.7) | <0.001 |
| *Mycoplasma genitalium*  Positive/Total | 25/400 (6.3) | 36/781 (4.6) | 16/361 (4.4) | NS |
| Yeasts  Positive/Total | 129/400 (32.3) | 256/781 (32.8) | 86/361 (23.8) | 0.006 |
