# Supplementary material for: The Complex Vaginal Flora of West African Women with Bacterial Vaginosis
Source: PLoS One. 2011 Sep 20;6(9):e25082. doi: 10.1371/journal.pone.0025082 (PMC3176826; doi:10.1371/journal.pone.0025082)
Supplement: Table S4 — Prevalence of micro-organisms according to self-defined occupation. (DOC) [file pone.0025082.s004.doc]

**Table S4. Prevalence of micro-organisms according to self-defined occupation.**

|  | Sex worker | | p-value |
| --- | --- | --- | --- |
|  | Yes | No |  |
| *Gardenerella vaginalis*  Positive/Total | 312/573 (54.5) | 523/969 (54.0) | NS |
| *Mycoplasma hominis*  Positive/Total | 151/573 (26.4) | 199/969 (20.5) | 0.01 |
| *Atopobium vaginae*  Positive/Total | 218/573 (38.0) | 397/969 (41.0) | NS |
| *Prevotella*  Positive/Total | 317/571 (55.5) | 401/969 (41.4) | <0.001 |
| *Mobiluncus*  Positive/Total | 36/571 (6.3) | 49/969 (5.1) | NS |
| *Eggerthella*  Positive/Total | 192/570 (33.7) | 237/966 (24.5) | <0.001 |
| *Megasphaera elsdenii*  Positive/Total | 91/570 (16.0) | 135/966 (14.0) | NS |
| *Leptotrichia*  Positive/Total | 257/570 (45.1) | 309/964 (32.1) | <0.001 |
| *Dialister*  Positive/Total | 166/570 (29.1) | 216/965 (22.4) | 0.004 |
| *Bifidobacterium*  Positive/Total | 389/570 (68.2) | 576/965 (59.7) | <0.001 |
| *Anaerococcus*  Positive/Total | 108/569 (19.0) | 196/965 (20.3) | NS |
| *Peptoniphilus* other than *lacrimalis*  Positive/Total | 138/569 (24.3) | 168/965 (17.4) | 0.002 |
| *Lactobacillus*  Positive/Total | 415/573 (72.4) | 769/969 (79.4) | 0.002 |
| *Trichomonas vaginalis*  Positive/Total | 93/573 (16.2) | 59/969 (6.1) | <0.001 |
| *Neisseria gonorrhoeae*  Positive/Total | 53/573 (9.2) | 14/969 (1.4) | <0.001 |
| *Chlamydia trachomatis*  Positive/Total | 41/573 (7.2) | 18/969 (1.9) | <0.001 |
| *Mycoplasma genitalium*  Positive/Total | 55/573 (9.6) | 22/969 (2.3) | <0.001 |
| Yeasts  Positive/Total | 136/573 (23.7) | 335/969 (34.6) | <0.001 |
