# Supplementary material for: The Complex Vaginal Flora of West African Women with Bacterial Vaginosis
Source: PLoS One. 2011 Sep 20;6(9):e25082. doi: 10.1371/journal.pone.0025082 (PMC3176826; doi:10.1371/journal.pone.0025082)
Supplement: Table S5 — Prevalence of micro-organisms according to number of sex partners in the last 3 months. (DOC) [file pone.0025082.s005.doc]

**Table S5. Prevalence of micro-organisms according to number of sex partners in the last 3 months.**

|  | Number of sex partners in last three months | | | p-value^1^ |
| --- | --- | --- | --- | --- |
|  | None | One | Two or more |  |
| *Gardenerella vaginalis*  Positive/Total | 83/180 (46.1) | 477/884 (54.0) | 272/472 (57.6) | 0.03 |
| *Mycoplasma hominis*  Positive/Total | 36/180 (20.0) | 200/884 (22.6) | 114/472 (24.2) | NS |
| *Atopobium vaginae*  Positive/Total | 65/180 (36.1) | 356/884 (40.3) | 191/472 (40.5) | NS |
| *Prevotella*  Positive/Total | 74/180 (41.1) | 379/883 (42.9) | 262/471 (55.6) | <0.001 |
| *Mobiluncus*  Positive/Total | 4/180 (2.2) | 59/883 (6.7) | 21/471 (4.5) | 0.03 |
| *Eggerthella*  Positive/Total | 41/179 (22.9) | 233/881 (26.4) | 154/470 (32.8) | 0.01 |
| *Megasphaera elsdenii*  Positive/Total | 22/179 (12.3) | 135/881 (15.3) | 69/470 (14.7) | NS |
| *Leptotrichia*  Positive/Total | 52/179 (29.1) | 300/880 (34.1) | 212/469 (45.2) | <0.001 |
| *Dialister*  Positive/Total | 39/179 (21.8) | 207/880 (23.5) | 135/470 (28.7) | NS |
| *Bifidobacterium*  Positive/Total | 99/179 (55.3) | 539/880 (61.3) | 324/470 (68.9) | 0.002 |
| *Anaerococcus*  Positive/Total | 34/179 (19.0) | 176/879 (20.0) | 93/470 (19.8) | NS |
| *Peptoniphilus* other than *lacrimalis*  Positive/Total | 37/179 (20.7) | 151/879 (17.2) | 115/470 (24.5) | 0.006 |
| *Lactobacillus*  Positive/Total | 152/180 (84.4) | 677/884 (76.6) | 350/472 (74.2) | 0.02 |
| *Trichomonas vaginalis*  Positive/Total | 10/180 (5.6) | 74/884 (8.4) | 67/472 (14.2) | <0.001 |
| *Neisseria gonorrhoeae*  Positive/Total | 6/180 (3.3) | 24/884 (2.7) | 37/472 (7.8) | <0.001 |
| *Chlamydia trachomatis*  Positive/Total | 2/180 (1.1) | 27/884 (3.1) | 30/472 (6.4) | 0.001 |
| *Mycoplasma genitalium*  Positive/Total | 2/180 (1.1) | 38/884 (4.3) | 37/472 (7.8) | <0.001 |
| Yeasts  Positive/Total | 59/180 (32.8) | 301/884 (34.0) | 109/472 (23.1) | <0.001 |

^1^ comparing distribution in all three columns
