# Supplementary material for: The Complex Vaginal Flora of West African Women with Bacterial Vaginosis
Source: PLoS One. 2011 Sep 20;6(9):e25082. doi: 10.1371/journal.pone.0025082 (PMC3176826; doi:10.1371/journal.pone.0025082)
Supplement: Table S6 — Prevalence of micro-organisms according to HIV status. (DOC) [file pone.0025082.s006.doc]

**Table S6. Prevalence of micro-organisms according to HIV status.**

|  | HIV status | | p-value |
| --- | --- | --- | --- |
|  | Negative | Positive |  |
| *Gardenerella vaginalis*  Positive/Total | 649/1236 (52.5) | 141/216 (65.3) | <0.001 |
| *Mycoplasma hominis*  Positive/Total | 261/1236 (21.1) | 70/216 (32.4) | <0.001 |
| *Atopobium vaginae*  Positive/Total | 493/1236 (39.9) | 89/216 (41.2) | NS |
| *Prevotella*  Positive/Total | 552/1234 (44.7) | 133/216 (61.6) | <0.001 |
| *Mobiluncus*  Positive/Total | 62/1234 (5.0) | 20/216 (9.3) | 0.02 |
| *Eggerthella*  Positive/Total | 340/1232 (27.6) | 72/214 (33.6) | NS |
| *Megasphaera elsdenii*  Positive/Total | 185/1232 (15.0) | 32/214 (15.0) | NS |
| *Leptotrichia*  Positive/Total | 439/1232 (35.6) | 99/214 (46.3) | 0.004 |
| *Dialister*  Positive/Total | 298/1231 (24.2) | 73/214 (34.1) | 0.003 |
| *Bifidobacterium*  Positive/Total | 751/1231 (61.0) | 160/214 (74.8) | <0.001 |
| *Anaerococcus*  Positive/Total | 246/1230 (20.0) | 44/214 (20.6) | NS |
| *Peptoniphilus* other than *lacrimalis*  Positive/Total | 225/1230 (18.3) | 64/214 (29.9) | <0.001 |
| *Lactobacillus*  Positive/Total | 965/1236 (78.1) | 150/216 (69.4) | 0.007 |
| *Trichomonas vaginalis*  Positive/Total | 107/1236 (8.7) | 34/216 (15.7) | 0.002 |
| *Neisseria gonorrhoeae*  Positive/Total | 39/1236 (3.2) | 27/216 (12.5) | <0.001 |
| *Chlamydia trachomatis*  Positive/Total | 48/1236 (3.9) | 10/216 (4.6) | NS |
| *Mycoplasma genitalium*  Positive/Total | 50/1236 (4.0) | 24/216 (11.1) | <0.001 |
| Yeasts  Positive/Total | 393/1236 (31.8) | 51/216 (23.6) | 0.02 |
