# Supplementary material for: The Complex Vaginal Flora of West African Women with Bacterial Vaginosis
Source: PLoS One. 2011 Sep 20;6(9):e25082. doi: 10.1371/journal.pone.0025082 (PMC3176826; doi:10.1371/journal.pone.0025082)
Supplement: Table S7 — Correlates of bacterial vaginosis (Nugent score ≥7) in multivariate analysis, excluding patients with an intermediate Nugent score (4–6). (DOC) [file pone.0025082.s007.doc]

**Table S7. Correlates of bacterial vaginosis (Nugent score ≥7) in multivariate analysis, excluding patients with an intermediate Nugent score (4-6).**

|  | Adjusted odds ratio (95% CI) |
| --- | --- |
| Number of sex partners, last 3 months  None  One  Two or more | 1.00  1.41 (0.84-2.37)  0.91 (0.52-1.60) |
| *Gardenerella vaginalis*  Negative  Positive | 1.00  2.67 (1.84-3.87)^1^ |
| *Bifidobacterium*  Negative  Positive | 1.00  2.50 (1.73-3.60)^1^ |
| *Megasphaera elsdenii*  Negative  Positive | 1.00  1.64 (0.94-2.86) |
| *Dialister*  Negative  Positive | 1.00  2.82 (1.61-4.96)^1^ |
| *Mycoplasma hominis*  Negative  Positive | 1.00  2.95 (1.76-4.97)^1^ |
| *Leptotrichia*  Negative  Positive | 1.00  2.03 (1.31-3.13)^1^ |
| *Prevotella*  Negative  Positive | 1.00  2.30 (1.58-3.33)^1^ |
| *Peptoniphilus* other than *lacrimalis*  Negative  Positive | 1.00  0.67 (0.41-1.08) |
| *Lactobacillus*  Negative  Positive | 1.00  0.20 (0.13-0.30)^1^ |

^1^ ≤0.001
