# Supplementary material for: The Complex Vaginal Flora of West African Women with Bacterial Vaginosis
Source: PLoS One. 2011 Sep 20;6(9):e25082. doi: 10.1371/journal.pone.0025082 (PMC3176826; doi:10.1371/journal.pone.0025082)
Supplement: Table S8 — Correlates of bacterial vaginosis (Nugent score ≥7) in multivariate analysis, excluding patients with an intermediate Nugent score (4–6). Presence of Lactobacillus has been removed from this model. (DOC) [file pone.0025082.s008.doc]

**Table S8. Correlates of bacterial vaginosis (Nugent score ≥7) in multivariate analysis, excluding patients with an intermediate Nugent score (4-6). Presence of Lactobacillus has been removed from this model.**

|  | Adjusted odds ratio (95% CI) |
| --- | --- |
| Number of sex partners, last 3 months  None  One  Two or more | 1.00  1.51 (0.92-2.47)  1.05 (0.62-1.80) |
| *Gardenerella vaginalis*  Negative  Positive | 1.00  1.89 (1.34-2.67)^1^ |
| *Bifidobacterium*  Negative  Positive | 1.00  3.04 (2.14-4.30)^1^ |
| *Megasphaera elsdenii*  Negative  Positive | 1.00  1.67 (0.97-2.85) |
| *Dialister*  Negative  Positive | 1.00  2.87 (1.65-4.98)^1^ |
| *Mycoplasma hominis*  Negative  Positive | 1.00  2.89 (1.73-4.82)^1^ |
| *Leptotrichia*  Negative  Positive | 1.00  2.23 (1.46-3.39)^1^ |
| *Prevotella*  Negative  Positive | 1.00  2.38 (1.67-3.40)^1^ |
| *Peptoniphilus* other than *lacrimalis*  Negative  Positive | 1.00  0.60 (0.37-0.96)^2^ |

^1^ ≤0.001; ^2^ ≤0.05
