# Supplementary material for: The Complex Vaginal Flora of West African Women with Bacterial Vaginosis
Source: PLoS One. 2011 Sep 20;6(9):e25082. doi: 10.1371/journal.pone.0025082 (PMC3176826; doi:10.1371/journal.pone.0025082)
Supplement: Table S9 — Proportion of patients with bacterial vaginosis who reported a complete resolution of vaginal discharge by Day 14. (DOC) [file pone.0025082.s009.doc]

**Table S9. Proportion of patients with bacterial vaginosis who reported a complete resolution of vaginal discharge by Day 14.**

|  | Treatment | | All |
| --- | --- | --- | --- |
|  | Metronidazole-clotrimazole | Tinidazole-Fluconazole |  |
|  | Complete response/Total (%) | Complete response/Total (%) | Complete response/Total (%) |
| Age, years  ≤20  21-30  ≥31 | 50/75 (67)  85/142 (60)  36/60 (60) | 46/72 (64)  89/147 (61)  50/74 (68) | 96/147 (65)  174/289 (60)  86/134 (64) |
| HIV  Negative  Positive | 131/212 (62)  34/50 (68) | 145/222 (65)  29/40 (73) | 276/434 (64)  63/90 (70) |
| *Gardenerella vaginalis*  Negative  Positive | 47/74 (64)  124/203 (61) | 41/74 (55)  144/219 (66) | 88/148 (59)  268/422 (64) |
| *Bifidobacterium*  Negative  Positive | 21/39 (54)  150/238 (63) | 33/53 (62)  152/240 (63) | 54/92 (59)  302/478 (63) |
| *Megasphaera elsdenii*  Negative  Positive | 122/203 (60)  49/74 (66) | 132/222 (59)  53/71 (75)^2^ | 254/425 (60)  102/145 (70)^2^ |
| *Dialister*  Negative  Positive | 96/164 (59)  75/113 (66) | 108/180 (60)  77/113 (68) | 204/344 (59)  152/226 (67) |
| *Mycoplasma hominis*  Negative  Positive | 107/174 (61)  64/103 (62) | 113/188 (60)  72/105 (69) | 220/362 (61)  136/208 (65) |
| *Leptotrichia*  Negative  Positive | 68/118 (58)  103/159 (65) | 71/132 (54)  113/159 (71)^2^ | 139/250 (56)  216/318 (68)^2^ |
| *Prevotella*  Negative  Positive | 53/98 (54)  118/179 (66) | 56/113 (50)  129/179 (72)^1^ | 109/211 (52)  247/358 (69)^1^ |
| Total number of BV-related micro-organisms  None  One  Two  Three  Four  Five  Six  Seven | 8/17 (47)  8/16 (50)  26/39 (67)  26/47 (55)  29/44 (66)  24/42 (57)  41/56 (73)  9/16 (56) | 5/15 (33)  16/26 (62)  22/47 (47)  23/38 (61)  39/53 (74)  36/52 (69)  30/46 (65)  13/13 (100)^2^ | 13/32 (41)  24/42 (57)  48/86 (56)  49/85 (58)  68/97 (70)  60/94 (64)  71/102 (70)  22/29 (76)^2^ |

^1^ ≤0.001  ^2^ ≤0.05
