# Supplementary material for: The Complex Vaginal Flora of West African Women with Bacterial Vaginosis
Source: PLoS One. 2011 Sep 20;6(9):e25082. doi: 10.1371/journal.pone.0025082 (PMC3176826; doi:10.1371/journal.pone.0025082)
Supplement: Text S1 — Methods used for nucleic acid amplification testing for various bacterial genus or species. (DOC) [file pone.0025082.s010.doc]

**Text S1. Methods used for nucleic acid amplification testing for various bacterial genus or species.**

Specimens in transport medium (Roche, Laval, Quebec) were treated with an equal volume of CT/NG specimen diluent (Roche). An equal volume (25µl) of treated sample was added to a PCR mix to give final concentrations of 1.25 U of HotStar Taq (Qiagen, Mississauga, Ontario), 3 mM MgCl2, 200 µM each dNTP (GE Healthcare, Baie d’Urfé, Quebec) and 0.2 µM (or 0.4 µM for *G. vaginalis*) of each of the six primers necessary to amplify targets in *G. vaginalis, A. vaginae* and *Lactobacillus* spp. After 15 minutes at 95°C, 40 cycles of 30 sec at 94°C, 30 sec at 60°C and 1 min at 72°C were performed and followed by 5 min at 72°C then cooled to 4°C. A 10 µl aliquot was then analyzed by electrophoresis on a 2% agarose gel and positive reactions were identified after staining with ethidium bromide. The same procedure was employed to simultaneously detect *Prevotella* spp. and *Mobiluncus* spp. using 0.4 µM of each primer, except that the PCR re-annealing step was at 55°C followed by 30 sec at 72°C. A similar strategy was employed to detect *M. hominis* except that home made Taq polymerase was employed in a mix containing 2 mM MgCl2 using specific primers and a different amplification program. After 5 minutes at 95°C, 40 cycles of 20 sec at 93°C, 20 sec at 55°C and 20 sec at 72°C were performed and followed by 5 min at 72°C before cooling to 4°C. Multiplex real-time PCR was used to simultaneously detect *Eggerthella, Leptotrichia* and *M. elsdenii*, or *Dialister* and *Bifidobacterium* or *Anaerococcus* and *Peptoniphilus* non-*lacrimalis*, using 0.4 µM of each primer and 0.1 µM probe with the LC480 Probe Master kit (Roche) on a Roche LC480 thermal cycler. Two µl of a mixture of equal volumes of specimen and specimen diluent were added to a final volume of 12 µl and, after 10 minutes at 95°C, 50 cycles of 10 sec at 93°C, 15 sec at 55°C and 15 sec at 72°C were performed. A similar strategy was employed to detect individually BVAB-1, BVAB-2, BVAB-3, *P. lacrimalis* and the *Clostridium coccoides* group using the LC480 SYBR Green I Master kit (Roche) followed by melting curve analysis. Published primer sequences [10, 15-20] and novel primers were evaluated on series of rDNA sequences gleaned from the Ribosomal Database Project II (http://rdp.cme.msu.edu/) using the Clustal alignment program (<http://www.pasteur.fr/seqanal/interfaces/clustalw.html>). Primer pairs were evaluated using clinical and ATCC bacterial isolates of *G. vaginalis, A. vaginae,* *Lactobacillus* spp, *M. hominis, Prevotella* spp., *Mobiluncus* spp., *Eggerthella, Leptotrichia*, *Bifidobacterium spp.,* and *Clostridium* spp.. The chosen PCR conditions were evaluated on 20 clinical specimens in comparison with Frederick’s conditions using agarose gel electrophoresis for BVAB-1, BVAB-2, BVAB-3, *P. lacrimalis, Peptoniphilus* non-*lacrimalis*. *A. vaginae,* and *M. elsdenii*, and in comparison with Matsuki’s conditions for *Prevotella* spp., *Bifidobacterium*, and the *Clostridium coccoides* group [10, 16].
